# Clinical features in different age groups of patients with autoimmune hepatitis

**DOI:** 10.3892/etm.2013.1363

**Published:** 2013-10-25

**Authors:** MILIN PENG, YI LI, MIN ZHANG, YONGFANG JIANG, YUAN XU, YI TIAN, FENG PENG, GUOZHONG GONG

**Affiliations:** Center of Liver Diseases, Second Xiangya Hospital, Central South University, Changsha, Hunan 410011, P.R. China

**Keywords:** autoimmune hepatitis, clinical presentation, concurrent autoimmune diseases, China

## Abstract

The Chinese population are at an increased risk of autoimmune hepatitis (AIH). The aims of this study were to determine the demographic and clinical features of AIH in China. A total of 83 patients with AIH diagnosed by the revised scoring system were re-analyzed, and the clinical presentations among the different ages were compared. The patients were classified according to age at presentation. AIH occurred in patients aged ≤30 years (9.6%), 31–39 years (10.8%), 40–49 years (16.9%), 50–59 years (31.3%) and ≥60 years (31.3%). There were no differences in the form of the clinical presentation, concurrent autoimmune diseases, cirrhosis distribution and autoantibodies among the groups. However, patients aged ≥60 years presented with higher levels of alkaline phosphatase (ALP) and γ-glutamyl transpeptidase (γ-GT) compared with patients aged ≤30 years (P=0.034, P=0.043, respectively), and patients aged 31–39 years had a significantly lower immunoglobulin G (IgG) level compared with those aged 50–59 years (P=0.049) and those aged ≥60 years (P=0.012). By contrast, patients aged ≤30 years had a significantly higher total bilirubin (TBIL) level compared with those aged 31–39 years (P=0.007), 50–59 years (P=0.002) and ≥60 years (P=0.013). A substantial portion of patients with AIH were aged >60 years, indicating a poor liver-associated outcome under current management strategies. Elderly patients appeared to be more asymptomatic compared with the younger patients.

## Introduction

Autoimmune hepatitis (AIH) is an unknown cause of liver inflammation, and the diagnosis of AIH requires the presence of characteristic clinical and laboratory features, and histological abnormalities (1). The incidence of AIH among white, Northern Europeans is 1.9 cases per 100,000 people per year, and its point prevalence is 16.9 cases per 100,000 people per year (2). In the USA, AIH affects between 100,000 and 200,000 individuals (3), and it accounts for 6% of liver transplantations (4). The frequency of AIH among patients with chronic liver disease in North America is between 11 and 23% (5). AIH should be considered in all individuals with acute and chronic hepatitis with an undetermined cause (6). Clinical presentations of the disease are variable, ranging from asymptomatic abnormal liver enzymes to fulminant liver failure or advanced decompensated cirrhosis (7). The frequency of cirrhosis at presentation is >30% (8). Concurrent immune disorders may mask the diagnosis of AIH. The diagnostic criteria and scoring system for AIH were codified by an international panel in 1993 (10) and revised in 1999 (1).

In comparison with the age- and gender-matched general population, with effective treatments available the mortality of AIH is two-fold higher than that of the general population (11,12). A long-term study reported that only ~40% of patients with AIH achieve complete remission (13). Traditionally, it was considered that clinical and demographic factors were closely associated with a poor outcome. Studies have reported that age distribution, serum aspartate aminotransferase (AST), alanine aminotransferase (ALT) and serum albumin levels were significant predictors of liver-associated mortality or liver transplantation (14,15). The majority of the current studies regarding the clinical characteristics associated with a poor outcome have focused on European and Japanese patients. Therefore, it is important to identify the clinical manifestations found in Chinese patients with AIH at various ages.

## Patients and methods

### Study population

Patients with AIH who were admitted to the Second Xiangya Hospital (Changsha, China) between 2002 and 2013, and who had complete clinical, laboratory and histological data, were enrolled in this retrospective study. The diagnoses were based on the 1999 revised criteria of the International Group of Autoimmune Hepatitis (1). Liver biopsy results were included if available, and two hepatopathologists reviewed all liver tissue specimens. Those patients with insufficient data for the diagnosis of AIH prior to treatment and other liver diseases [viral hepatitis, Wilson’s disease, non-alcoholic fatty liver disease (NFLD), primary biliary cirrhosis (PBC) and primary sclerosing cholangitis (PSC)] were excluded from this study. Patients were categorized according to their age at presentation into the following groups: ≤30 years, 31–39 years, 40–49 years, 50–59 years and ≥60 years. This study was approved by The Ethics Committee of the Second Xiangya Hospital of Central South University (Changsha, China). Informed consent was obtained from all patients.

### Clinical and laboratory assessments

Clinical examinations and conventional laboratory tests were assessed prior to providing any specific therapy to the patients. The presence of other concurrent autoimmune diseases was also investigated. Liver function tests were performed, including the measurements of the serum levels of alkaline phosphatase (ALP), γ-glutamyl transpeptidase (γ-GT), AST, ALT, total bilirubin (TBIL) and serum immunoglobulin G (IgG), by performing immunonephelometry on all patients. Smooth muscle antibodies (SMA), antinuclear antibodies (ANA), perinuclear antineutrophil cytoplasmic antibodies (pANCA), antimitochondrial antibodies (AMA), antibodies to liver kidney microsome type 1 (LKM1) and soluble liver antigen/liver-pancreas antigen (SLA/LP) were evaluated using indirect immunofluorescence. Each patient was seronegative for AMA.

### Statistical analysis

A descriptive analysis, including means, standard deviation and frequencies, is presented. An independent sample t-test was used to assess the correlation between continuous variables and the χ^2^ test was used for the analysis of categorical variables. P<0.05 was considered in indicate a statistically significant difference. The SPSS statistical software version 17.0 (SPSS, Inc., Chicago, IL, USA) was used for all statistical analyses.

## Results

### Study population

**Figure 1:**
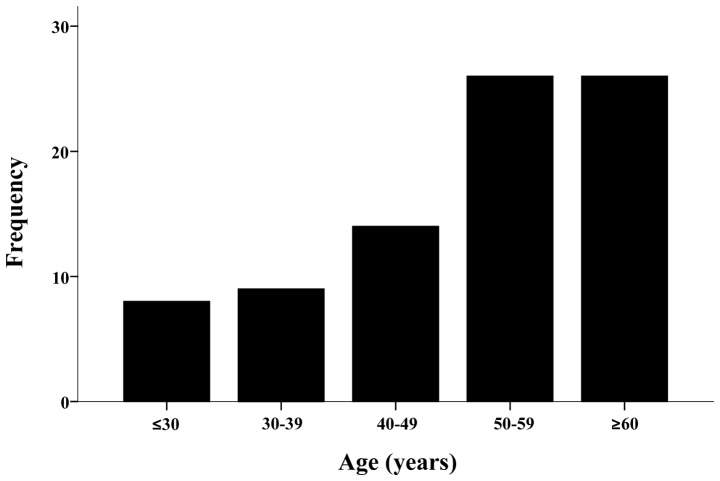
Age distribution of autoimmune hepatitis in 83 Chinese patients.

A total of 83 Chinese patients fulfilled the 1999 revised criteria of the International Group of Autoimmune Hepatitis; 41 patients (49.4%) were classified as ‘definite’ AIH and 42 (50.6%) were classified as ‘probable’ AIH. At presentation, the patients were graded according to their age. Patients aged ≤30 years (9.6%), 31–39 years (10.8%), 40–49 years (16.9%), 50–59 years (31.3%) and ≥60 years (31.3%) with AIH were enrolled in the study. In total, 26 patients (31.3%) were ≥60 years old. The mean age of this group (the elderly population) was 65.62±4.92 years (range, 60–78 years) and the male to female ratio was 3:23. In total, 26 patients (31.3%) were aged between 50 and 59 years. The mean age was 54.65±2.67 years and the male to female ratio was 2:24. A total of eight patients (9.6%) constituted the young population, with the mean age of 21.38±6.74 years (range, 10–30 years). The disease frequency in each age range increased >40 years (Fig. 1), and the peak occurrences were in the patients aged 50–59 years and in the elderly patients. Each group was similar with regard to gender distribution.

### Clinical presentation

The clinical manifestations of AIH are unspecific, and other chronic liver diseases may have the same symptoms. Anorexia, fatigue and jaundice were the most common symptoms observed in >70% of the 83 patients. Furthermore, 15% presented with a fever and ~10% of patients had pruritus. Overall, 76.9% of patients presented with up to three symptoms, and 23.1% presented with four symptoms. Liver cirrhosis was diagnosed in 10 (12%) patients, among whom five patients (50%) were between the ages of 50 and 59 years and three patients (30%) were elderly. However, the frequency of cirrhosis was not statistically different between the elderly patients (≥60 years old) and the young patients (≤30 years old) (P>0.05).

### Laboratory data

The mean serum levels of liver function are illustrated in Table I. Patients aged ≤30 years (136.53±74.03 U/l, P=0.034) and aged 40–49 years (147.43±52.71 U/l, P=0.021) presented with significantly lower levels of of ALP than patients aged ≥60 years (238.78±163.29 U/l). Patients aged ≤30 years (119.71±145.92 U/l) also presented with significantly lower γ-GT levels compared with patients aged 50–59 years (257.69±175.56 U/l, P=0.037) and those aged ≥60 years (253.27±193.96 U/l, P=0.043). Similarly, patients aged 31–39 years (18.74±5.83 g/l) presented with significantly lower IgG levels than those aged 50–59 years (25.41±6.05 g/l, P=0.049) and those aged ≥60 years (27.29±11.33 g/l, P=0.012). By contrast, patients aged ≤30 years (351.33±284.94 μmol/l) had significant higher TBIL levels compared with those aged 31–39 years (116.46±106.09 μmol/l, P=0.007), those aged 50–59 years (125.28±112.43 μmol/l, P=0.002) and those aged ≥60 years (172.83±33.82 μmol/l, P=0.013).

In total, 51 of the 83 patients were positive for ANA with a titer of ≥1:40. Among the elderly patients, 19 were positive for ANA, while among the patients aged 50–59 years and ≤30 years, 17 and five patients were positive for ANA, respectively. However, there were no differences in the positive proportions of ANA among each group (Table I).

### Concurrent autoimmune diseases

Of the 26 elderly patients, 10 (59%) presented with coexisting autoimmune diseases, including four patients with Sjögren’s Syndrome, five patients with systemic lupus erythematosus and one patient with ulcerative colitis. Similarly, 11 of the 26 patients aged 50–59 years presented with concurrent autoimmune diseases (four patients with Sjögren’s Syndrome, two with autoimmune thyroiditis and rheumatoid arthritis, and three with systemic lupus erythematosus). Five of the eight younger patients (aged ≤30 years) presented with concurrent autoimmune diseases (two patients with autoimmune thyroiditis and systemic lupus erythematosus, and one patient with rheumatoid arthritis). However, the distribution of concurrent autoimmune diseases in each age group was not statistically significant (P=0.398).

## Discussion

In China, the etiology of chronic hepatitis is mainly hepatitis virus infection. Since the recognition of autoimmune liver diseases, these diseases have become one of the major forms of non-viral chronic liver diseases in China. Studies on the prevalence of Chinese patients with AIH are rare.

The present study investigated the differences associated with age in the clinical and laboratory features of Chinese patients with AIH diagnosed using the revised 1999 scoring system (1). In this study, the age of onset in Chinese patients showed that the peak occurrences of AIH exist in the age groups of 50–59 years and ≥60 years, and there are few young patients (9.6%) with AIH. The proportion of young patients is smaller than in previous studies regarding Japanese patients. The frequency of liver cirrhosis at first diagnosis of AIH was <12%, lower than other studies where cirrhosis at presentation was detected and reported in 27–42% of patients (16,17).

The present study demonstrated that elderly patients (≥60 years old) presented with higher serum ALP and γ-GT levels compared with younger patients (≤30 years old). By contrast, the young patients presented with higher TBIL levels compared with elderly patients. This result indicated that patients aged ≤30 years may have more severe disease activity than elderly patients. A previous study from Japan demonstrated that elderly patients presented with more asymptomatic clinical features compared with the young patients (17). The results of the present study also suggested that elderly patients may be misdiagnosed in the early stage due to the fact that they may not exhibit any symptoms. This may be one of the reasons for the prevalence of autoimmune diseases and the increased presentation in elderly patients compared with younger patients in China. However, the occurrence of cirrhosis in each group was similar in the present study, and the rate was markedly lower compared with groups of patients from North America (16) and Japan (17).

AIH is associated with a number of distinct circulating autoantibodies. The most common autoantibodies are ANA and SMA. It was revealed that 96% of adult patients with AIH from North America had ANA and SMA (18). Anti-SLA/LP and pANCA may be useful in identifying patients with AIH from those who lack typical serological findings (19,20). Anti-SLA/LP are highly specific markers of AIH and more commonly found in association with the conventional autoantibodies; however, they also are occasionally found in patients with AIH who are negative for ANA, SMA, and anti-LKM1 (19). It has been reported that anti-SLA are highly specific for the diagnosis of autoimmune liver disease, and their detection may reveal patients with a more severe degree of the disease and a worse predicted outcome (21). pANCA are non-specific, but are commonly present. They have been used to reclassify patients with cryptogenic chronic hepatitis, such as AIH; however, they have not been formally assimilated into the diagnostic algorithm (20). In the present study, we observed that ANA was the most common autoantibody in Chinese patients with AIH. Among these Chinese patients with AIH, 61.4% were ANA-positive. SLA/LP (14.5%) was another common autoantibody. Serum levels of AST and IgG were found to be higher in ANA-positive patients; however, there was no statistically significant difference between patients who were positive and negative for ANA, which was similar to a previous Japanese study (22).

Concurrent immune disorders may mask the underlying liver disease. Autoimmune thyroiditis, Graves’ disease, synovitis and ulcerative colitis are the most common immune-mediated disorders associated with AIH in adults from North America (23). Notably, concurrent immune diseases were more commonly observed in this group of Chinese patients with AIH (42.2%) than in the group of patients from the west (24). It has been reported that associated autoimmune diseases were observed in 15–34% of patients from the West (24). In the present study it was observed that Sjögren’s Syndrome was the most commonly associated autoimmune disorder. According to a nationwide survey in Japan (22), the prevalence of complicating Sjögren’s syndrome in patients with AIH was ~10%. Czaja and Carpenter (16) reported that elderly patients presented with concurrent thyroid and rheumatic diseases more often than patients aged ≤30 years. Certain studies have suggested that human leukocyte antigen DR4 occurred more frequently in elderly patients. However, in the present study, autoimmune thyroiditis was uncommon in elderly Chinese patients. The distribution of the human leukocyte antigen is not fully recognized in China, and further studies are required to analyze the human leukocyte antigen in large number of patients with AIH.

A long-term study established that incomplete normalization of ALT at 6 months, low serum albumin concentration at diagnosis, and an age at presentation of <20 years or >60 years were significant independent predictors of a poor outcome (15). However, histological cirrhosis at diagnosis was not associated with a poor prognosis and did not affect the response to initial immunosuppressive treatment. In the present study, a considerable portion of patients with AIH were aged >60 years, indicating a poor liver-associated outcome under current management strategies. In addition, even when treated with the usual management strategy, there is an increased risk that extra-hepatic malignancy, such as skin (non-melanoma) and hematological cancers, may be induced by immunosuppressive therapy. Further studies should explore effective ways to improve the survival of patients with AIH without increasing the risk of extra-hepatic malignancy.

One of the limitations of the present study was the relatively small number of patients; multicenter national data are required. Moreover, the detection of HLA-DR3 and DR4 is rare in China, and the results from the studies performed in other countries have demonstrated that HLA status may affect clinical manifestations, behavior and the outcome of treatment (16).

In conclusion, a substantial portion of patients with AIH were >60 years old, indicating a poor liver-associated outcome under current management strategies. Elderly patients exhibited higher levels of ALP and γ-GT, and a lower TBIL level than younger patients. Chinese patients with AIH had a lower frequency of cirrhosis at presentation and a higher frequency of concurrent autoimmune diseases compared with patients from Europe, North America and Japan. However, the frequencies of cirrhosis, concurrent autoimmune disease and autoantibodies were similar among each group.

## Figures and Tables

**Table I:** Clinical features of 83 Chinese patients with autoimmune hepatitis in each group.

	Age category (years) (n=83)				
	
Features	≤30 (n=8)	31–39 (n=9)	40–49 (n=14)	50–59 (n=26)	≥60 (n=26)
Female (n)	5	6	13	24	23
Age (years)	21.3±6.7	37.4±1.9	45.9±2.8	54.6±2.6	65.6±4.9
Cirrhosis (n)	1	1	0	5	3
ALT (U/l)	151.8±133.7	312.2±338.4	288.5±233.5	384.2±397.7	287.9±288.4
AST (U/l)	193.6±147.9	422.9±596.9	353.0±314.1	385.7±367.1	310.6±213.1
TBIL (μmol/l)	351.3±284.9	116.5±106.1	203.5±224.9	125.3±112.4	172.2±172.8
ALP (U/l)	136.5±74.0	187.5±138.5	147.4±52.7	191.2±83.4	238.8±163.3
γ-GT (U/l)	119.7±145.9	161.5±95.1	135.8±81.7	257.7±175.6	253.3±194.0
IgG (g/l)	25.1±11.4	18.7±5.8	22.1±6.1	25.4±6.1	27.3±11.3
ANA (≥1:40)	5	3	7	17	19

ALT, alanine aminotransferase; AST, aspartate aminotransferase; TBIL, total bilirubin; ALP, alkaline phosphatase; γ-GT, γ-glutamyl transpeptidase; IgG, immunoglobulin G; ANA, antinuclear antibodies.
